# SOX9 expression decreases survival of patients with intrahepatic cholangiocarcinoma by conferring chemoresistance

**DOI:** 10.1038/s41416-018-0338-9

**Published:** 2018-11-13

**Authors:** Xiaodong Yuan, Jun Li, Cédric Coulouarn, Tao Lin, Laurent Sulpice, Damien Bergeat, Carolina De La Torre, Roman Liebe, Norbert Gretz, Matthias P. A. Ebert, Steven Dooley, Hong-Lei Weng

**Affiliations:** 1 Department of Medicine II, Medical Faculty Mannheim, Heidelberg University, Mannheim, Germany; 2 Department of General Visceral Transplantation, University Hospital Tübingen, Tübingen, Germany; 3 Inserm, Inra, University Rennes, UMR 1241, Nutrition Metabolisms and Cancer (NuMeCan), Service de Chirurgie Hépatobiliaire et Digestive, Rennes, France; 4 Medical Research Center, Medical Faculty of Mannheim, University of Heidelberg, Mannheim, Germany; 5 Department of Medicine II, Saarland University Medical Center, Saarland University, Homburg, Germany

**Keywords:** Bile duct cancer, Chemotherapy

## Abstract

**Background:**

Sex-determining region Y-box (SRY-box) containing gene 9 (SOX9) expression confers cancer stem cell features. However, SOX9 function in intrahepatic cholangiocarcinoma (iCCA) is unknown. This study investigated the effects and underlying mechanisms of SOX9 in iCCA.

**Methods:**

SOX9 expression in 59 iCCA patients was examined by immunohistochemistry. The association between SOX9 expression and clinical outcome was evaluated. Gene signature and biological functions of SOX9 in iCCA were examined in vitro.

**Results:**

iCCA patients with high SOX9 expression had shorter survival time than those with low SOX9. In patients receiving chemotherapy, median survival time in patients with low and high levels of SOX9 were 62 and 22 months, respectively. In vitro, gemcitabine increased SOX9 expression in iCCA cells. When SOX9 was knocked down, gemcitabine-induced apoptosis was markedly increased. Silencing SOX9 significantly inhibited gemcitabine-induced phosphorylation of checkpoint kinase 1, a key cell cycle checkpoint protein that coordinates the DNA damage response and inhibited the expression of multidrug resistance genes. Microarray analyses showed that SOX9 knockdown in CCA cells altered gene signatures associated with multidrug resistance and p53 signalling.

**Conclusions:**

SOX9 governs the response of CCA cells to chemotherapy. SOX9 is a biomarker to select iCCA patients eligible for efficient chemotherapy.

## Introduction

Cholangiocarcinoma (CCA) is the second most common primary liver cancer following hepatocellular carcinoma (HCC), and accounts for approximately 10–15% of all primary liver malignancies.^1^ The global incidence and mortality rate for CCA have been increasing over the past decades.^2,3^ Anatomically, CCA is classified into intrahepatic (iCCA) and extrahepatic cholangiocarcinoma (eCCA) depending on the location of the tumour along the biliary tract.^4^ To date, curative surgical resection is the most efficient treatment for long-term survival of selected iCCA patients.^5,6^ However, in most cases, the tumours are quite advanced at the time of diagnosis and surgical resection is not possible.^5^ Systemic chemotherapy and radiotherapy regimens remain the only approach to render patient eligible for surgery and palliative treatment.^7^ However, the response of iCCA to these treatments is very weak.^7^ Therefore, elucidating the underlying mechanisms of iCCA chemoresistance is one key issue to improve survival of patients.

Sex-determining region Y-box (SRY-box) containing gene 9 (SOX9) belongs to the SOX family of transcription factors.^8^ It is widely expressed in multiple organs during embryonic development, including the liver.^9,10^ In liver embryogenesis, SOX9 expression is the most specific and earliest marker of hepatoblasts and determines the timing of intrahepatic bile duct morphogenesis.^11,12^ In normal adult liver, SOX9 is expressed in the periportal small intrahepatic ducts, and peribiliary glands lining the large bile ducts.^13^ SOX9 plays important roles in maintaining liver homeostasis, regulating liver regeneration, and eventually in liver cancer development.^14^ In acute or chronic liver disease, SOX9 expression robustly manifests in ductular reactions (DRs), which contain putative progenitor cells capable of differentiating into both cholangiocytes and hepatocytes.^15^ Moreover, SOX9-positive cells express stem cell markers, such as epithelial cell adhesion molecule (EpCAM), neural cell adhesion molecule, CD133 and CXC motif chemokine receptor 4.^13,16^ In contrast to normal hepatocytes, where SOX9 is not expressed, a subset of HCC cells displayed SOX9 expression. These patients usually demonstrate severe venous cancer invasion, advanced tumour stage and shorter survival.^17^ Recent studies reported that SOX9-positive HCC cells exhibit liver cancer stem cell (CSC)-like features, and that SOX9 in cancer cells confers self-renewal and tumourigenicity by promoting symmetrical cell division.^17,18^ To date, only few studies had addressed the role of SOX9 in CCA.^19^ Here, we report clinical and functional data supporting an oncogenic role and therapeutic significance of SOX9 expression in iCCA.

## Materials and methods

### Patients and liver tissues

This study enroled 59 iCCA patients from Tübingen, Germany (18 iCCA patients) and Rennes, France (41 iCCA patients) between 2002 and 2010. In addition, 21 liver tissues from patients with chronic hepatitis B infection were enroled in Mannheim, Germany. Basic characteristics of the enroled chronic hepatitis B and iCCA patients are shown in Supplementary Tables 1 and 2. The study protocol fulfilled national laws and regulations and was approved by the local Ethics Committees.

### Cell culture and treatment

The following cell lines were investigated in the study: CC-SW-1 and HuCCT-1 (iCCA lines), EGI-1 and TFK-1 (eCCA lines), HCCC-9810 (mix CCA line) and MMNK-1 (normal cholangiocyte line). EGI-1, CC-SW-1 and MMNK-1 were cultured in Dulbecco's modified Eagle's medium (DMEM) (BE12-709F, Lonza) supplemented with 10% foetal bovine serum (FBS) (10270-098, Invitrogen), 4mM l-glutamine (17-605C, Lonza) and 100 U/mL penicillin/streptomycin (A2210 Biochrom KG). TFK-1 and HuCCT-1 were cultured in RPMI-1640 supplemented with 10% FBS (10270-098, Invitrogen), 4 mM l-glutamine (17-605 C, Lonza) and 100 U/mL penicillin/streptomycin (A2210 Biochrom KG). All the cell lines were cultured in a humidified incubator at 37 °C and with 5% CO_2_ atmosphere.

Cells underwent starvation without FBS medium for 10 to 16 h before treatment with gemcitabine and cisplatin (kindly provided by Prof. Lu LG, Shanghai Jiao Tong University School of Medicine), which was dissolved in phosphate-buffered saline (PBS) to make a 100 mM stock solution and diluted with cell culture medium to indicated concentrations during treatment.

### Immunohistochemistry and staining evaluation

Tissue microarray assay was performed as previously described.^20^ In brief, formalin-fixed, paraffin-embedded specimens were deparaffinised in serial ethanol dilutions and rehydrated. After a single PBS wash, heat-induced antigen retrieval was performed with 1 mM EDTA solution, pH 8.4 (03677; Sigma-Aldrich, Steinheim, Germany) at 98 °C for 10 min. Endogenous peroxidase activity was blocked with Dako dual endogenous enzyme blocking reagent (S2003; Dako, Via Real, Carpinteria, CA, USA), followed by blocking with 3% hydrogen peroxidase for 5 min at room temperature to prevent unspecific binding of antibodies. The tissue sections were incubated with polyclonal rabbit anti-SOX9 antibody (HPA001758; Sigma-Aldrich) at a dilution of 1:100, or monoclonal mouse anti-cytokeratin 19 (CK19) antibody (SC-6287; Santa Cruz) at a dilution of 1:100 overnight at 4 °C. The specimens were subsequently washed in PBS for 3×5 min and incubated with anti-rabbit or anti-mouse secondary antibody conjugated with horse radish peroxidase (HRP) for 1 h at room temperature, and then detected with 3,3′-diaminobenzidine for 7 min. The slides were counterstained with haematoxylin. All sections were dehydrated and mounted with malinol mounting medium.

Immunostaining results for SOX9 were scored semi-quantitatively based on the intensity and proportion of positive tumour cell nuclei. In detail, the intensity score of SOX9 nuclear staining was defined as four grades: 0, negative; 1, weak with colour yellow; 2, medium with colour brown; 3, strong with colour black. The number of cells with SOX9-positive nuclei was defined as six grades: 0, no detectable positive cells; 1, positive cells ≤1%; 2, positive cells >1%, and ≤10%; 3, positive cells >10%, and ≤33%; 4, positive cells > 33%, and ≤66%; 5, positive cells >66%. The final immune staining scores were calculated as the intensity scores × the proportion scores. The samples with final scores over 10 were defined as “high SOX9 expression”, and the remainder as “low SOX9 expression”. The representative pictures of SOX9 staining and for semi-quantitative scoring system are presented in Supplementary Figure 1. CK19 expression was categorised into high expression and low expression according to the immunoreactivity in tumour cells. The immunoreactivity of CK19 was defined as four grades: 0, positive cells ≤1%; 1, positive cells >1% and ≤33%; 2, positive cells >33% and ≤66%; 3, positive cells >66%. The samples with grade 3 were defined as high CK19 expression, and the others were low CK19 expression.

### RNA interference

For transient transfection of short interfering RNA (siRNA), cells were treated with indicated culture medium without penicillin/streptomycin. siRNA targeting human SOX9 (M-021507-00) and control siRNA (D-001206-14) were purchased from Dharmacon. SOX9 siRNA were transfected with RNAiMAX (13778, Invitrogen). The transfection was performed in six-well cell culture vessels. Tumour cells were plated at a density of 1.5 × 10^5^ cells per well with 2 mL corresponding growth medium. Briefly, for siRNA transfection, 2 μl RNAiMAX was mixed with 20 pmol SOX9 siRNA in 200 μl Opti-MEM medium. The mixtures were preincubated for 20 min at room temperature before adding to cells. RNA and whole-cell proteins were extracted 48 and 60 h after transfection for further examination, respectively.

### MTT assay

Cells were incubated with 5 mg/mL 3-(4, 5-dimethylthiazolyl-2)-2, 5-diphenyltetrazolium bromide (MTT) reagent (M5655, Sigma-Aldrich) for 5 h. Then, the supernatant was removed carefully and the 100 µL solvent solution containing 40 µL of 10% sodium dodecyl sulphate (SDS), 40 µL dimethyl sulfoxide and 20 µL of 1.2% acetate acid solution (600 µL acetate acid in 50 mL PBS) was added and incubated overnight for measurement. Absorbance was measured at 570 nm with a reference to 630 nm. For cell viability assay and gemcitabine half-maximal inhibitory concentration (IC_50_) measurement, cells were incubated in 96-well plate for 48 h before incubation with MTT.

### Cell cycle analysis

Cells were harvested at 48 h after siRNA treatment and washed with cold PBS, and then fixed with 70% cold ethanol. To remove RNA, the cells were re-suspended in solution containing Triton X-100 (0.1%) and 100 µg/mL RNase. The samples were stained with propidium iodide (20 μg/mL) for 30 min in the dark, and then subjected to analysis for DNA content using FACS Calibur (BD Biosciences, Heidelberg, Germany) and data analysis was performed using FlowJo version10 software.

### Transwell migration assay

Cell culture inserts with 8 μM pore size (Falcon) were used. For tumour cell migration, 2.0 × 10^5^ iCCA tumour cells were suspended in RPMI or DMEM medium with 0.5% FBS and plated in the upper chambers. The lower chambers were filled RPMI or DMEM with 10% FBS. After 16 h, the medium in the inserts were removed and washed with PBS. The inserts were filled with 3.7% formaldehyde for 5 min. Thereafter, the inserts were incubated in methanol for 30 min. The filters were stained with 10% Giemsa (Sigma, St. Louis, MO, USA) for 15 min. The inner side was wiped with cotton swabs. Migrated cells were counted under a light microscope.

### Caspase-3 assay

Caspase-3 assay was performed as previously described.^21^ In brief, cells were lysed in 80 µL of lysis buffer (50 mM HEPES, 100 mM NaCl, 0.1% CHAPS, 1 mM DTT, 0.1 mM EDTA, pH 7.4). Then, 20 μl of cell lysate were incubated in 70 μl reaction buffer (50 mM HEPES, 100 mM NaCl, 0.1% CHAPS, 10 mM DTT, 0. mM EDTA, 10% (w/v) glycerol, pH 7.4) and 10 μl AC-DEVD-AFC caspase-3 fluorimetric substrate (Biomol, Hamburg, Germany) for 90 min at 37 °C. Subsequently, caspase-3 activity was detected by fluorometric measurement using Tecan infinite M200 (excitation 400 nm; emission 505 nm). The caspase-3 activity was normalised to protein levels and reported as relative fluorescent units per minute per mg protein.

### Immunoblotting

Immunoblotting assay was performed as previously described.^20^ Briefly, total cell protein was extracted on ice using radio immunoprecipitation assay buffer with freshly added protease and phosphatase inhibitors. Protein concentrations were assessed with a Bio-Rad protein assay. Twenty micrograms of total cell protein extracts was subjected to 10% or 12% SDS-polyacrylamide electrophoresis gel and transferred to nitrocellulose membranes. Five per cent non-fat milk in Tris-buffered saline with Tween-20 (TBST) was used to block nonspecific binding. Membranes were probed with primary and secondary antibodies in TBST according to the manufacturer’s instructions. HRP-linked anti-mouse and anti-rabbit Abs were used as secondary antibodies. α-Tubulin and glyceraldehyde 3-phosphate dehydrogenase were used as a loading control. Signal was visualised by incubating the blots in Supersignal Ultra (Pierce, Hamburg, Germany).

### RNA isolation and quantitative real-time reverse transcription polymerase chain reaction

Total cell RNA was extracted using the InviTrap Spin Universal RNA Mini Kit (Stratec, Berlin, Germany), according to the manufacturer’s instructions. For first-strand complementary DNA (cDNA) synthesis, reverse transcription of 500 ng RNA was performed with random primers (Thermo Scientific) and RevertAid H Minus M-MuLV reverse transcriptase (Thermo Scientific) according to the manufacturer’s instructions and subsequently diluted with nuclease-free water (Invitrogen) to 10 ng/µL cDNA. For PCR amplification, 10.4 µL mixtures contained 5 µL (50 ng) template cDNA, 5 µL SYBR Green (4367659, Life Technologies) and 4 µM forward and reverse primer PCRs were run in triplicate and performed on a StepOnePlus Real-time PCR (Applied Biosystems). PCR amplification cycling conditions comprised 10 min polymerase activation at 95 °C and 40 cycles at 95 °C for 15 s and 60 °C for 1 min. A melting-curve analysis was performed for each PCR analysis. Relative quantification of target genes was normalised against the housekeeping gene *PPIA*.

#### Microarray

Gene expression profiling was performed using arrays of human HuGene-2_0-st-type from Affymetrix. Biotinylated antisense cDNA was prepared according to the Affymetrix standard labelling protocol with the GeneChip^®^ WT Plus Reagent Kit and the GeneChip^®^ Hybridization, Wash and Stain Kit (both from Affymetrix, Santa Clara, CA, USA). Subsequently, the hybridisation on the chip was performed on a GeneChip Hybridization oven 640, then dyed in the GeneChip Fluidics Station 450 and thereafter scanned with a GeneChip Scanner 3000. All of the equipment used was from the Affymetrix-Company (Affymetrix, High Wycombe, UK).

#### Bioinformatic analyses

A Custom CDF Version 21 with ENTREZ-based gene definitions was used to annotate the arrays.^22^ The Raw fluorescence intensity values were normalised applying quantile normalisation and RMA background correction. Differential expressed genes were identified by using a commercial software package SAS JMP10 Genomics, version 6, from SAS (SAS Institute, Cary, NC, USA). A false-positive rate of *a* = 0.05 with false discovery rate correction was taken as the level of significance.

### Statistical analyses

Variables were summarised as means ± standard deviation (SD) and depicted graphically as means ± SD. *P* values were calculated using the *χ*^2^ test or calculated using a two-sided (unpaired) Student’s *t* test. Kaplan–Meier survival curve and univariate Cox analysis was used to evaluate overall survival (OS) rates and disease-free survival (DFS) rate of iCCA patients. *P* values were calculated using the log-rank test. *P* < 0.05 was considered significant.

## Results

### SOX9 has distinct expression patterns in chronic liver disease and CCA

**Fig. 1:**
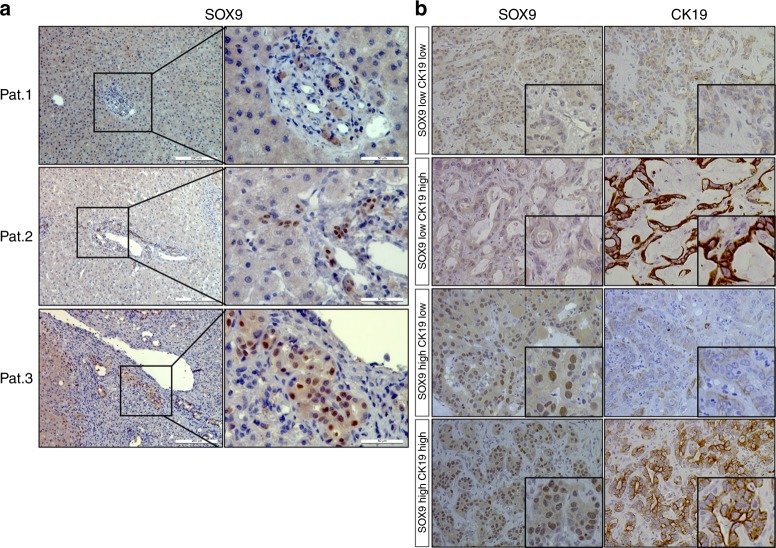
Expression of SOX9 and CK19 in patients with iCCA. **a** SOX9 express in cells of bile ducts (Patient 1) and reactive ducts (Patient 2) of liver tissues with chronic HBV infection. Patient 3 shows negative SOX9 immune reaction in the liver. **b** Four patterns of SOX9 and CK19 expression in iCCA patients

First, we compared expression of SOX9 and CK19, two classic markers of biliary tree, in 80 patients with chronic liver disease or iCCA. Among 21 patients with chronic liver disease, 17 showed SOX9-positive immunoreactivity, whereas 4 were negative (Fig. 1a shows representative patients). In contrast to SOX9, CK19 immunostaining was positive in all patients (data not shown). The results suggest that SOX9 expression in cholangiocytes is unstable in chronic liver disease compared to CK19. Distinct from CK19, which localised in the cytoplasm of cholangiocytes, SOX9 was expressed in the nuclei of cells in the canals of Hering, reactive ductules and bile ducts (Patients 1 and 2, Fig. 1a). As in chronic liver disease, SOX9 expression was observed in the nuclei of iCCA tumour cells, while CK19 localised in the cytoplasm of cancer cells (Fig. 1b). Expressions of SOX9 and CK19 in cancer cells were heterogeneous. Figure 1b display four patterns of SOX9 and CK19 expression in iCCA: SOX9^high^CK19^high^, SOX9^high^CK19^low^, SOX9^low^CK19^high^ and SOX9^low^CK19^low^. There was no significant correlation between expression of SOX9 and CK19 in iCCA tumour cells (*P* > 0.05). In all examined tissue specimens, neither SOX9 nor CK19 were detected in hepatocytes.

### Expression of SOX9 predicts poor clinical outcome of iCCA

**Fig. 2:**
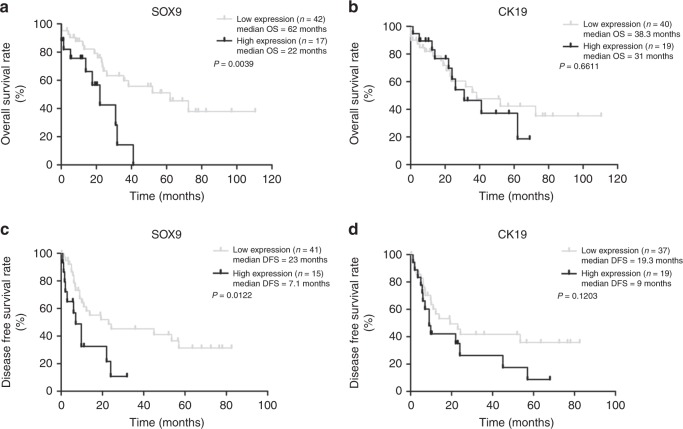
SOX9, but not CK19, is associated with survival time of iCCA patients. Kaplan–Meier survival analysis shows the association between SOX9 (**a**, **c**)/CK19 expression (**b**, **d**) and survival time in 59 iCCA patients.

Next, we analysed the correlation of SOX9 and CK19 expression with clinical parameters of the iCCA patients, including age, gender, vascular invasion, existence of cirrhosis and American Joint Committee on Cancer (AJCC) classification. Among these clinical parameters, CK19 expression was associated with AJCC classification of CCA, while SOX9 did not show any association with these clinical parameters (Supplementary Table 3). However, multivariate analysis showed that among the analysed variables, only SOX9 expression significantly influenced the over survival (OS) of iCCA patients (hazard ratio = 3.614, 95% confidence Interval = 1.493–9.076, *P* = 0.006, Supplementary Table 4). Furthermore, Kaplan–Meier analysis and the log-rank test showed that patients with high SOX9 expression had shorter OS and disease free survival (DFS) rates than those with low SOX9 expression (*P* < 0.01 and *P* < 0.05, respectively, Fig. 2a, c). The median OS time in patients with SOX9 low expression was 62 months, whereas the value in those patients with high SOX9 expression was only 22 months (Fig. 2a). In contrast to SOX9, there was no association between CK19 expression and survival time in these patients (*P* > 0.05, Fig. 2b, d).

**Fig. 3:**
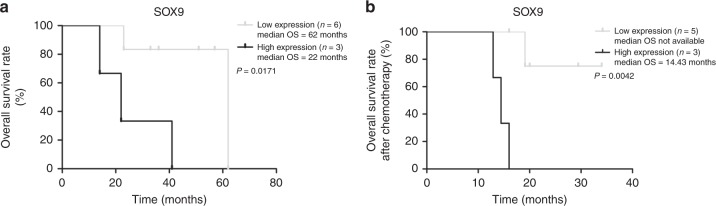
SOX9 is associated with survival time of iCCA patients receiving chemotherapy. **a** Kaplan–Meier plot for OS rate of nine iCCA patients received chemotherapy before or after cancer recurrence. **b** Kaplan–Meier plot for OS rate of eight iCCA patients received chemotherapy after cancer recurrence.

This cohort of iCCA included 9 patients who received chemotherapy (e.g. gemcitabine and cisplatin) (Supplementary Table 5). Among them, six patients had low SOX9 expression and three patients showed high levels of SOX9. Survival analyses revealed that patients with high SOX9 expression had shorter OS time (*P* < 0.05, Fig. 3a). In patients who received chemotherapy, the mean survival time in patients with SOX9 low expression was 62 months, whereas the value in those patients with high SOX9 expression was only 22 months (Fig. 3a). Except one patient who received chemotherapy following surgery, additional eight patients, five with low and three with high SOX9 expression, received chemotherapy due to the recurrence of iCCA. The survival times until the end of the follow-up in five patients with low SOX9 levels were 16, 19, 20, 29 and 34 months, whereas the values in 3 with high SOX9 expression were 13, 14 and 16 months, respectively (Supplementary Table 5). Survival difference between the two groups was significant (*P* < 0.01, Fig. 3a). Notably, four out of five patients with low SOX9 were still surviving when the follow-up ended (Supplementary Table 5). However, all three patients with high SOX9 expression were dead during follow-up (Supplementary Table 5). CK19 expression did not show any correlation with chemotherapy response (*P* > 0.05, data not shown).

### SOX9 inhibition sensitises CCA cells to gemcitabine

To investigate how SOX9 expression in CCA cell might modify their response to chemotherapy, we performed microarray analysis in iCCA CC-SW-1 cells after SOX9 silencing. We found that gene expression associated with drug metabolism and ABC transporters such as *ABCB1* (MDR1) and *ABCC4* (MRP4) was decreased, while genes related with the p53 signalling pathway were increased when SOX9 was knocked down with siRNA (Supplementary Figure 2A). Western blot and quantitative polymerase chain reaction (qPCR) further confirmed that the expression of multidrug resistance genes *ABCC4* and *ABCB1* was markedly reduced when SOX9 was inhibited in CC-SW-1 cells (Supplementary Figure 2B–C).

**Fig. 4:**
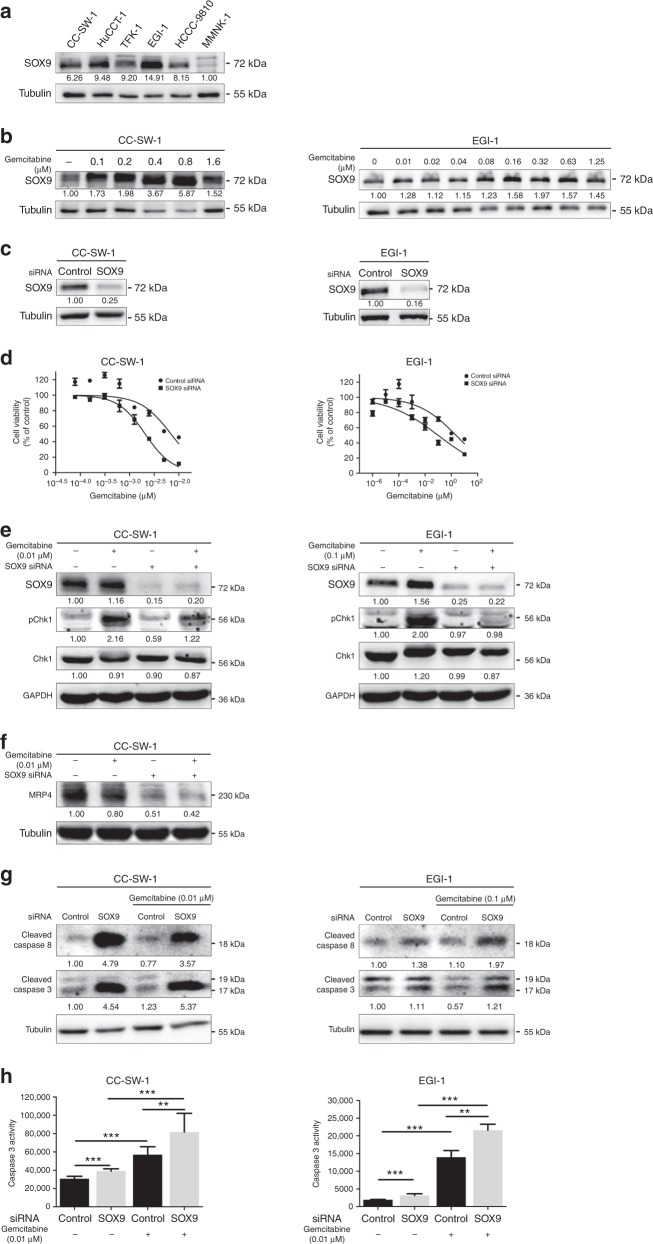
Disruption of SOX9 increases gemcitabine-induced iCCA cell apoptosis. **a** The expression of SOX9 in normal cholangiocytes and different CCA cells. **b** Administration of gemcitabine dose-dependently induced expression of SOX9 in both CC-SW1 and EGI-1 cells. **c** SOX9 was knocked down by siRNA. **d** MTT analyses showed cell viability in gemcitabine-treated CC-SW1 and EGI-1 cells with or without SOX9 knockdown. **e**–**g** Phosphorylation of CHK1, MRP4, cleaved caspase-3 and caspase-8 were measured in gemcitabine-treated CC-SW1 and EGI-1 cells with or without SOX9 knockdown. **h** Caspase-3 assay was used to measure caspase-3 activity in gemcitabine-treated CC-SW1 and EGI-1 cells with or without SOX9 knockdown. All western blot analyses in **a**–**c** and **e**–**g** were performed at least for three times. Quantification of western blot is shown by the numbers between bands

Next, we treated different types of CCA cells with gemcitabine, an analogue of deoxycytidine, which is widely used in the treatment of CCA. Notably, basal expression of SOX9 in CCA cells was significantly higher than in normal cholangiocytes (Fig. 4a). More impressively, expression of SOX9 protein was further increased upon gemcitabine treatment in both iCCA CC-SW-1 and eCCA EGI-1 cells (Fig. 4b). To examine the function of SOX9 in gemcitabine-treated CCA cells, we knocked down SOX9 expression using siRNA in CC-SW-1 and EGI-1 cells, followed by treatment with gemcitabine for 24 h (Fig. 4c). MTT assay showed that when SOX9 expression was inhibited, the IC_50_ of gemcitabine-treated cells significantly decreased from 7.1 ± 0.15 to 2.0 ± 0.23 nM in CC-SW-1 cells and from 380.3 ± 249.1 to 46.3 ± 21.9 nM in EGI-1 cells, respectively (Fig. 4d).

Given the key role of phosphorylation of checkpoint kinase 1 (CHEK1) in coordinating the DNA damage response and inhibiting the expression of multidrug resistance genes,^23^ we examined whether disruption of SOX9 impacted CHEK1 activation. Immunoblot analysis showed that SOX9 siRNA remarkably inhibited gemcitabine-dependent pCHEK1 in both CC-SW-1 and EGI-1 cells and MRP4 expression in CC-SW-1 cells (Fig. 4d–f). Consistent with reduced expression of MRP4 and pCHK1, analyses based on immunoblot and cleaved caspase-3 activity assay revealed marked increases in cleaved caspase-3 and caspase-8 expression and caspase activity in CCA cells with SOX9 knockdown, indicating that gemcitabine-induced apoptosis was increased when SOX9 expression was inhibited (Fig. 4g, h).

In addition to gemcitabine, we examined the role of SOX9 in cisplatin-treated CC-SW-1 and EGI cells. MTT assay showed that knockdown of SOX9 did not have impact on cisplatin-inhibited cell viability in both cells (Supplementary Figure 3A–B). In contrast to gemcitabine, administration of cisplatin and/or knockdown of SOX9 did not influence the expression of pCHEK1 (Supplementary Figure 3C–D).

### SOX9 is essential for CCA cell proliferation, stemness and migration

**Fig. 5:**
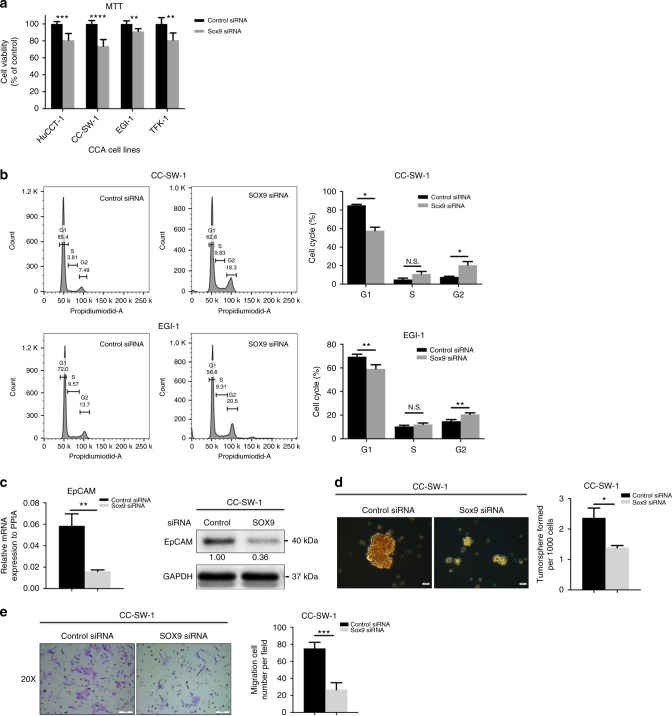
Disruption of SOX9 impact biological behaviours of CCA cells. **a** MTT analyses were performed to measure cell viability in different CCA cells with or without SOX9 knockdown. **b** FACS analyses were used to examine the impact of SOX9 on cell cycle of CC-SW1 and EGI-1 cells. **c** mRNA and protein expression of EpCAM were measured by qPCR and western blot in CC-SW1 and EGI-1 cells with or without SOX9 knockdown. **d** Tumour sphere formation assay was used to assess self-renewal of CC-SW-1 cells with or without SXO9 knockdown. **e** The impact of SOX9 on CC-SW-1 cell migration was examined with transwell migration assay. NS not significant; *, P<0.05; **, P<0.01, ****, P<0.0001

Next, we examined the role of SOX9 in CCA cell proliferation, stemness and migration. MTT analyses showed that knockdown of SOX9 expression significantly inhibited cell proliferation in four types of CCA cells (Fig. 5A). In CC-SW-1 and EGI-1 cells, SOX9 inhibition significantly decreased the proportion of cells staying in G1 phase and increased those in G2/M phase (Fig. 5b). The results suggest that SOX9 is required for maintaining CCA cell proliferation.

Subsequently, we investigated the effects of SOX9 on stemness of CCA cells. We found that knockdown of SOX9 expression decreased EpCAM expression at both RNA and protein levels in CC-SW-1 cells (Fig. 5c). In HCC, EpCAM is considered as a crucial factor in the maintenance of CSC-like features in cancer cells.^24^ To investigate whether SOX9 is implicated to the CSC features of CCA cells, we performed tumour sphere formation assay, a widely recognised method to evaluate cancer stem cell self-renewal and differentiation at the single-cell level in vitro.^25^ SOX9 knockdown significantly inhibited the capacity of tumour sphere formation in CC-SW-1 (Fig. 5d).

In addition, we also investigated the role of SOX9 in CCA cell migration. Transwell assay showed that knockdown of SOX9 expression significantly inhibited cell migration in CC-SW-1 cells (Fig. 5e).

## Discussion

**Fig. 6:**
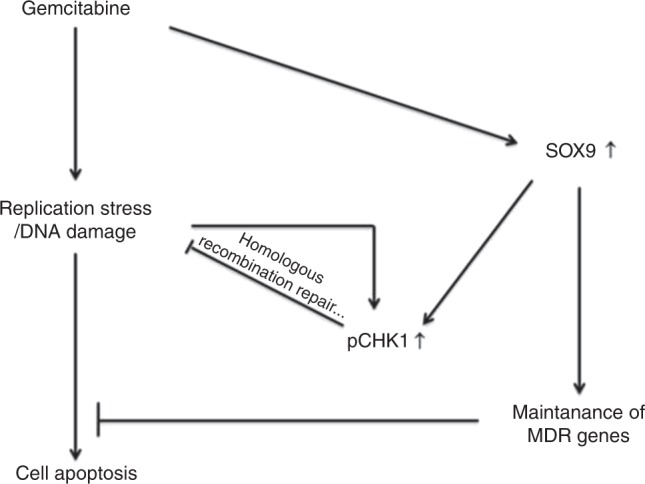
A scheme depicts the mechanisms on how SOX9 prevent cells from apoptosis in gemcitabine-treated CCA cells

The standard treatment for advanced-stage iCCA is systemic chemotherapy with gemcitabine and cisplatin.^7^ However, the median OS time is <12 months.^7^ In patients treated with gemcitabine alone, the survival time is <8 months,^7^ thus improving the sensitivity of cholangiocarcinoma cells to chemotherapy is a key to prolonging the survival of iCCA patients. In the current study, we found that SOX9, the earliest cholangiocyte marker during embryonic liver development,^11,12^ plays a crucial role in iCCA cells’ resistance to chemotherapy. We examined the expression of SOX9 in 59 iCCA patients who received surgery. High expression of SOX9 in the nuclei of iCCA cancer cells was significantly associated with shorter survival time (*P* = 0.0039). Of nine patients treated with chemotherapy following surgery, the median survival time reached 62 months in six patients who had low levels of SOX9 expression, whereas survival time was only 22 months in the three patients who had high SOX9 levels. Although the sample size was small in this study, the difference in survival time between both groups was significant (*P* = 0.017). Among the nine patients, eight received chemotherapy because of the recurrence of cancer. The five patients with low SOX9 levels survived between 16 and 34 months; however, the longest survival time in three patients with high SOX9 expression was only 16 months. These results suggest that SOX9 expression correlates with cholangiocellular cancer cells’ response to chemotherapy. Further in vitro studies provided the following mechanistic explanations of the observed differences:^1^ Microarray, qPCR and western blot analyses showed that disruption of SOX9 with siRNA significantly decreased expression of genes/proteins associated with drug metabolism and multidrug resistance and increased the abundance of genes associated with p53 signalling pathway.^2^ Knockdown of SOX9 markedly inhibited gemcitabine-induced activation of CHK1, a key cell cycle checkpoint protein that coordinates the DNA damage response, and expression of multiple drug resistance protein MRP4, and thus increased cancer cell apoptosis.^3^ Gemcitabine dose-dependently induced expression of SOX9, indicating that CCA cells increase SOX9 as a defensive mechanism against treatment with chemotherapeutics such as gemcitabine. Figure 6 depicts the gemcitabine-induced loop in CCA cells.

The role of SOX9 in the chemoresistance has been reported in multiple tumours, for example, chondrosarcoma, breast cancer, glioblastoma, cervical cancer, gastric cancer and lung cancer.^26–31^ In cervical cancer cells, SOX9 was found to increase cancer cell chemoresistance through inhibiting miR-130a.^27^ However, the detailed mechanisms of how SOX9 contributes to chemoresistance have not been clarified to date. Our observation that SOX9 confers chemoresistance to cholangiocarcinoma through the activation of CHK1 and the expression of multiple drug resistance proteins might provide a light pointer for further investigation of this aspect.

The current study also investigated the role of SOX9 in CCA cells receiving cisplatin. In contrast to gemcitabine, knockdown of SOX9 did not impact the efficiency of cisplatin. Administration of cisplatin did not alter expression of pChk1 in CCA cells. The discrepancy between the two compounds might be due to their different mechanisms of action. Gemcitabine results in cell death mainly through the inhibition of DNA synthesis and the inhibition of enzymes relevant to deoxyribonucleotide metabolism.^32^ Thus, gemcitabine exerts these actions through impacting multiple pathways, including regulating checkpoint kinases. Different from gemcitabine, cisplatin causes cell death through the formation of [PtCl(guanine-DNA)(NH3)2]+, which inhibits DNA repair and activates apoptosis.^33^

The defensive effects of SOX9 for CCA cells are not limited to cancer cells facing chemotherapy. Knockdown of SOX9 in both iCCA and eCCA cells remarkably inhibited the capacity of cancer cell proliferation and migration, decreased CSC stemness and increased apoptosis. These results provide an explanation why SOX9 expression is associated with survival time of patients receiving chemotherapy, but also in those who are treated by surgery alone. On the other hand, it should be kept in mind that these in vitro findings are not always consistent with the observation obtained in patients. For example, there was no correlation between the expression of SOX9 and cell proliferation markers, for example, Ki67 and PCNA, in iCCA patients (data not shown). Given that there are multiple growth factors and proliferative signals governing cancer cell expansion in patients with iCCA, it is not surprising that low levels of SOX9 expression alone does not have a significant impact on the proliferation of cancer cells. In addition, although there is a crucial role of SOX9 in maintaining cell identity, we did not observe a significant correlation between SOX9 expression and histological differentiation in this cohort of iCCA patients (data not shown). The result might have two explanations:^1^ SOX9 expression does not impact on differentiation of iCCA, and^2^ SOX9 might have a subtle influence on cancer cell differentiation, but the association is too small to be detected in the currently small number of specimens.

In diseased liver, high levels of SOX9 occur not only in CCA but also in HCC. However, in contrast to cholangiocytes, normal hepatocytes do not express SOX9. Expression of SOX9 in HCC reflects a cancer stem cell/progenitor cell.^18^ Given that SOX9 is the earliest and dominant phenotype marker of normal cholangiocytes, the induction of a cancer stem cell-like phenotype should not be attributed to the expression of SOX9. However, the current results do suggest that SOX9 plays a role in the maintenance of cancer stem cell phenotypes. Like CCA patients, HCC patients with high levels of SOX9 had poor prognosis. As in CCA, SOX9 in HCC is implicated in maintaining proliferation and self-renewal of cancer cells.^18^ In the future, it will be interesting to find out whether SOX9-dependent control of checkpoint protein activation may also play a role in chemoresistance of HCC.

Besides SOX9, this study analysed the association between CK19 expression and the clinical outcome of iCCA. CK19 is a classical marker for cholangiocytes. It has been reported that CK19 contributes to the differentiation of iCCA from metastatic adenocarcinoma and is associated with the histological differentiation of iCCA.^34^ In the current cohort of iCCA patients, CK19 expression was correlated with AJCC classification of iCCA, whereas it did not show any association with the survival of iCCA patients.

Taken together, SOX9 expression is a sensitive marker that predicts the survival time of iCCA patients, particularly in those receiving chemotherapy. Our study demonstrates that SOX9 is a key transcription factor that prevents iCCA cells from apoptosis when the cells are attacked by drugs such as gemcitabine. SOX9 exerts the observed effects on CCA cells, at least in part, through the activation of Chk1 and upregulation of multidrug resistance genes. Limitations of this study are: (1) the low number of samples precludes more general conclusions; (2) it is not clear how SOX9 exerts the observed effects on the expression of multidrug resistance genes; (3) we cannot conclude whether SOX9 expression has a similar effect in eCCA. Our study also enroled five eCCA patients who demonstrate a similar biological behaviour as iCCA (data not shown). Results in eCCA cell line EGI-1 indicate that the SOX9 expression has similar effects as observed in iCCA. Further investigation based on a large size of patient cohorts is required in the future.

## Electronic supplementary material

Author change letter

Supplementary Figure 1

Supplementary Figure 2

Supplementary Figure 3

Supplementary Table 1

Supplementary Table 2

Supplementary Table 3

Supplementary Table 4

Supplementary Table 5
