# Supplementary figures and images for: SOX9 expression decreases survival of patients with intrahepatic cholangiocarcinoma by conferring chemoresistance
Source: Br J Cancer. 2018 Nov 13;119(11):1358–66. doi: 10.1038/s41416-018-0338-9 (PMC6265288; doi:10.1038/s41416-018-0338-9)

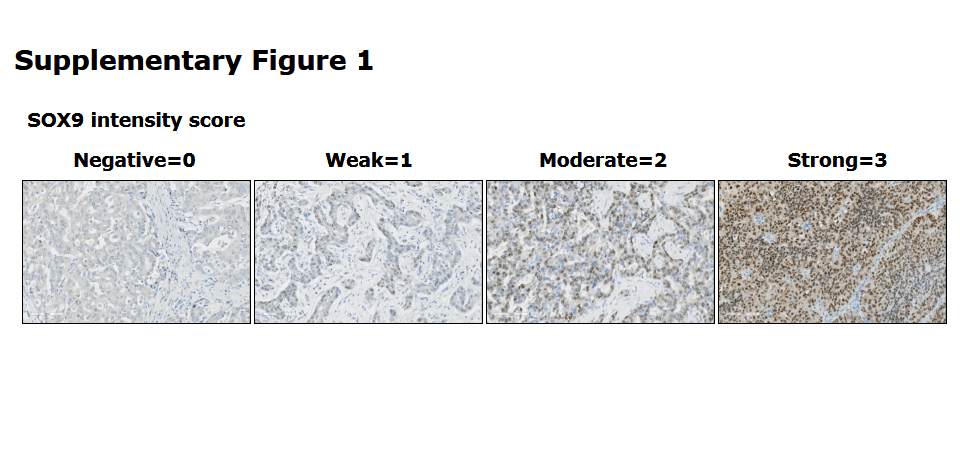

Supplement: Supplementary file 1 — Author change letter [file 41416_2018_338_MOESM1_ESM.tif]

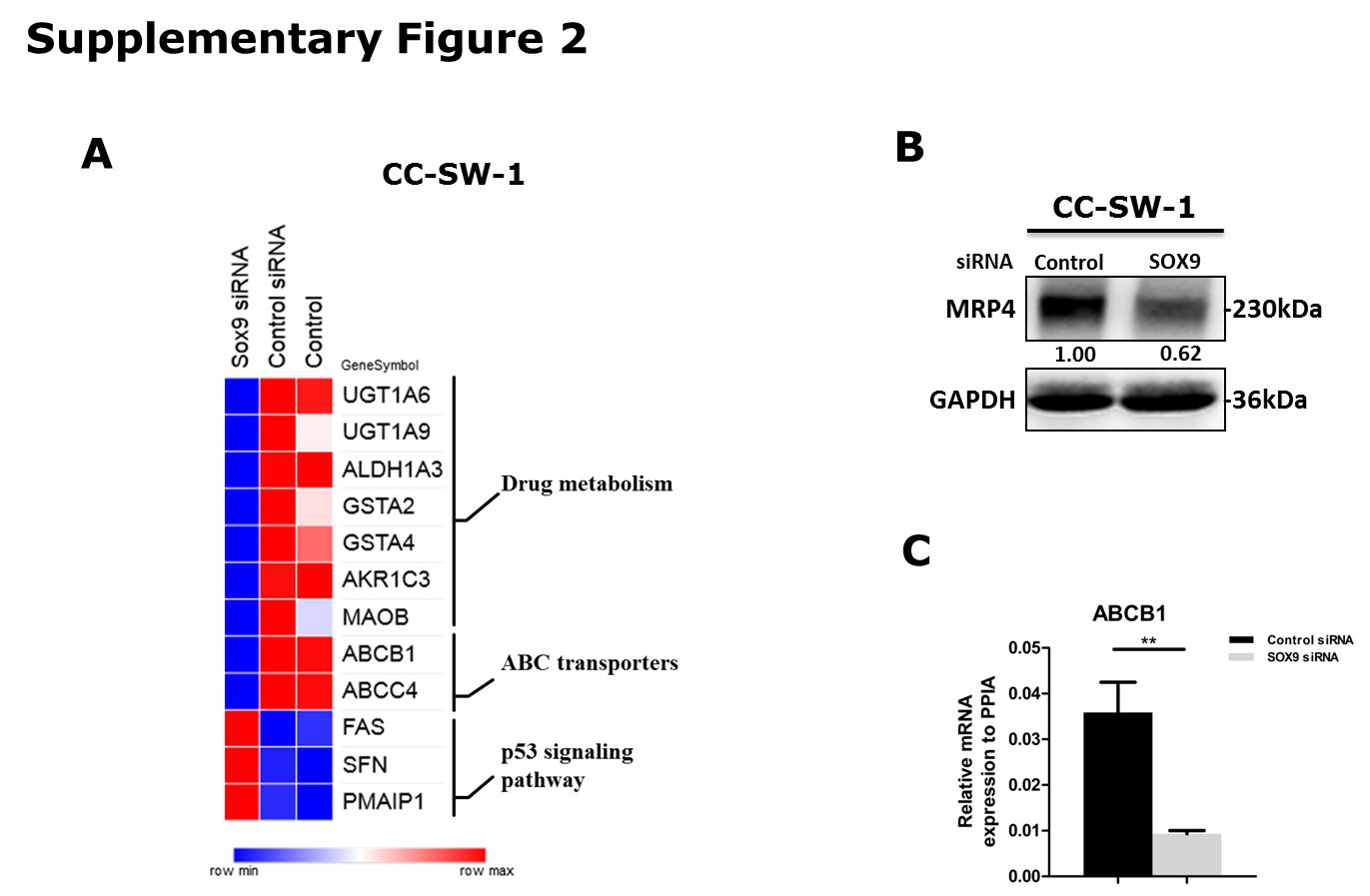

Supplement: Supplementary file 2 — Supplementary Figure 1 [file 41416_2018_338_MOESM2_ESM.jpg]

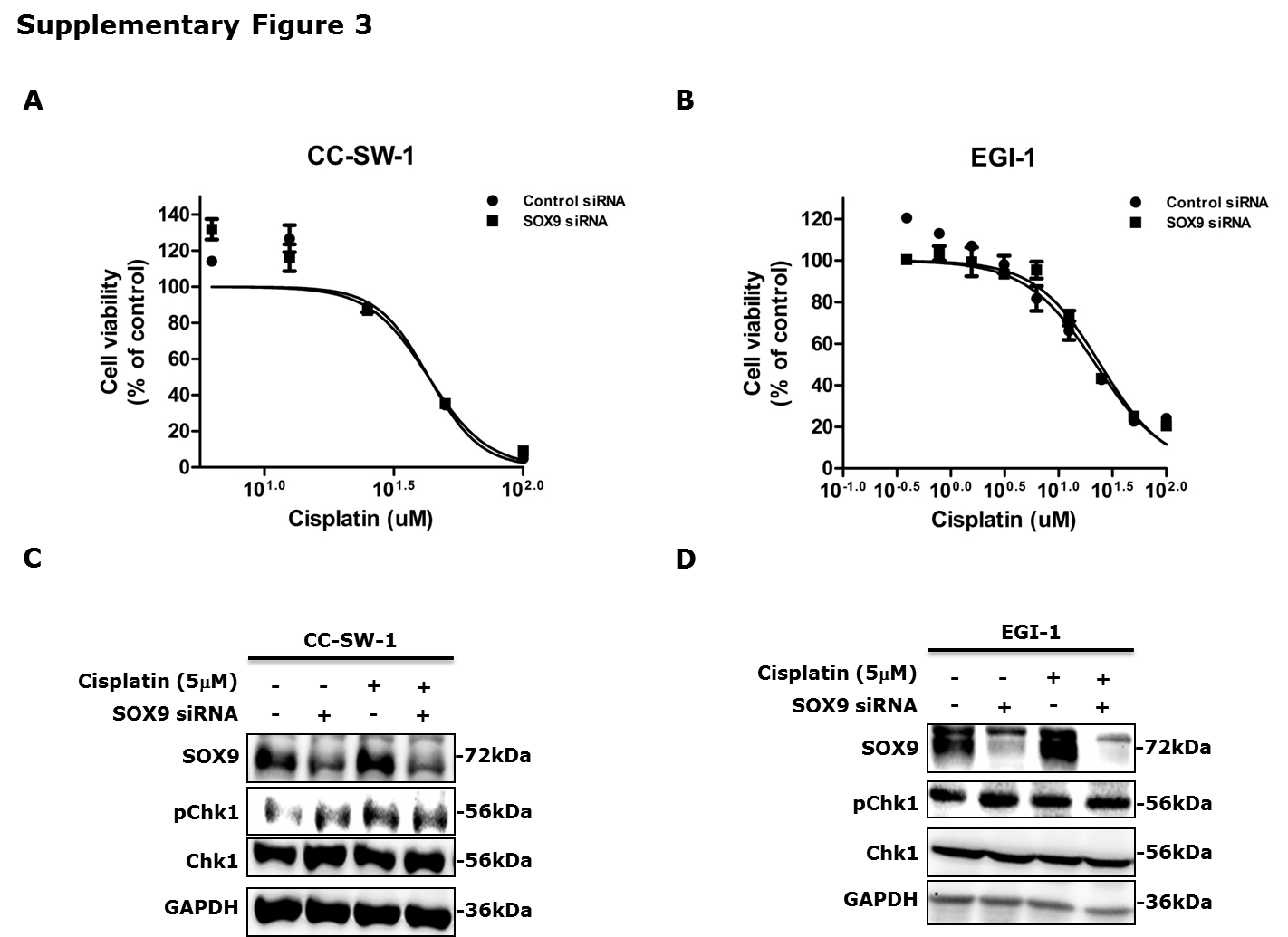

Supplement: Supplementary file 3 — Supplementary Figure 2 [file 41416_2018_338_MOESM3_ESM.tif]
