# Supplementary material for: SOX9 expression decreases survival of patients with intrahepatic cholangiocarcinoma by conferring chemoresistance
Source: Br J Cancer. 2018 Nov 13;119(11):1358–66. doi: 10.1038/s41416-018-0338-9 (PMC6265288; doi:10.1038/s41416-018-0338-9)
Supplement: Supplementary file 4 — Supplementary Figure 3 [file 41416_2018_338_MOESM4_ESM.docx]

**Supplementary Table 1.** Clinicopathological features of patients with chronic liver disease

| **Clinicopathological Features** | **N=21** |
| --- | --- |
| Age (years) | 19 - 65 |
| Gender (male/female) | 18/3 |
| Inflammatory grade* |  |
| 0 | 3 |
| 1 | 5 |
| 2 | 5 |
| 3 | 5 |
| 4 | 3 |
| Fibrotic Stage* |  |
| 0 | 3 |
| 1 | 3 |
| 2 | 5 |
| 3 | 4 |
| 4 | 6 |

*Inflammation and fibrosis was assessed according to Scheuer criterion.
