# Supplementary material for: SOX9 expression decreases survival of patients with intrahepatic cholangiocarcinoma by conferring chemoresistance
Source: Br J Cancer. 2018 Nov 13;119(11):1358–66. doi: 10.1038/s41416-018-0338-9 (PMC6265288; doi:10.1038/s41416-018-0338-9)
Supplement: Supplementary file 5 — Supplementary Table 1 [file 41416_2018_338_MOESM5_ESM.docx]

**Supplementary Table 2.** Clinicopathological features of validating set iCCA

| **Clinicopathological Features** | **N=59** |
| --- | --- |
| Age (years) | 63.47 ± 9.96 |
| Gender (male/female) | 44/15 |
| Vascular invasion | 8 (13.6%) |
| Cirrhosis | 18 (30.5%) |
| AJCC classification* |  |
| I | 21 (35.6%) |
| II | 18 (30.5%) |
| III | 9 (15.2%) |
| IV | 11 (18.6%) |
| Follow up (months) | 28.91 ± 26.60 |
| Death | 28 (47.5%) |

*AJCC 7th Edition of TNM Staging.
