# Supplementary material for: SOX9 expression decreases survival of patients with intrahepatic cholangiocarcinoma by conferring chemoresistance
Source: Br J Cancer. 2018 Nov 13;119(11):1358–66. doi: 10.1038/s41416-018-0338-9 (PMC6265288; doi:10.1038/s41416-018-0338-9)
Supplement: Supplementary file 6 — Supplementary Table 2 [file 41416_2018_338_MOESM6_ESM.docx]

**Supplementary Table 3**. Patient characteristics in iCCA patients with different levels of SOX9/CK19

| **Clinicopathological factors** | | **SOX9 expression** | | | | | **CK19 expression** | | | |
| --- | --- | --- | --- | --- | --- | --- | --- | --- | --- | --- |
|  |  | **low expression** **(n=42)** | | **high expression** **(n=17)** | | ***P* value** | **low expression** **(n=40)** | **high expression** **(n=19)** | | ***P* value** |
| **Age (year)** | | 64.55 ± 9.60 | | 60.82 ± 10.62 | | 0.20 | 60.00 ± 10.70 | 65.13 ± 9.30 | | 0.064 |
|  | **60>** | 12 | | 7 | | 0.34 | 10 | 9 | | 0.086 |
|  | **≥60** | 30 | | 10 | |  | 30 | 10 | |  |
| **Gender** | |  | | |  | |  | |  | |
|  | **male** | 30 | | 14 | | 0.58 | 31 | 13 | | 0.454 |
|  | **female** | 12 | | 3 | |  | 9 | 6 | |  |
| **Vascular invasion** | |  | | | | |  | | | |
|  | **Yes** | 4 | 4 | | | 0.32 | 5 | 3 | | 0.730 |
|  | **No** | 38 | 13 | | |  | 35 | 16 | |  |
| **Cirrhosis** | |  | | | | |  | | | |
|  | **Yes** | 11 | 7 | | | 0.26 | 11 | 7 | | 0.466 |
|  | **No** | 31 | 10 | | |  | 29 | 12 | |  |
| **AJCC classification** | |  | | | | |  | | | |
|  | **I+II** | 26 | 13 | | | 0.44 | 30 | 9 | | 0.036 |
|  | **II+III** | 16 | 4 | | |  | 10 | 10 | |  |
