# Supplementary material for: SOX9 expression decreases survival of patients with intrahepatic cholangiocarcinoma by conferring chemoresistance
Source: Br J Cancer. 2018 Nov 13;119(11):1358–66. doi: 10.1038/s41416-018-0338-9 (PMC6265288; doi:10.1038/s41416-018-0338-9)
Supplement: Supplementary file 7 — Supplementary Table 3 [file 41416_2018_338_MOESM7_ESM.docx]

Supplementary Table 4. Multivariate Analysis for Overall Survival of iCCA patients

| **Variable** | **Hazard Ratio** | **95% Confidence Interval** | ***P* Value** |
| --- | --- | --- | --- |
| **Age** | 1.049 | 1.000-1.100 | 0.048 |
| **Gender _(Male_ *_vs._* _Female)_** | 0.554 | 0.182-1.689 | 0.299 |
| **Cirrhosis _(Yes_ *_vs._* _No)_** | 1.086 | 0.494-2.391 | 0.837 |
| **AJCC _(I+II_ *_vs._* _III-IV)_** | 1.955 | 0.778-4.193 | 0.154 |
| **Vascular Invasion _(Yes_ *_vs._* _No)_** | 0.633 | 0.132-3.038 | 0.568 |
| **SOX9 _(High_ *_vs._* _Low)_** | **3.614** | **1.493-9.076** | **0.006** |
| **CK19 _(High_ *_vs._* _Low)_** | 0.949 | 0.383-2.348 | 0.910 |
