# Supplementary material for: SOX9 expression decreases survival of patients with intrahepatic cholangiocarcinoma by conferring chemoresistance
Source: Br J Cancer. 2018 Nov 13;119(11):1358–66. doi: 10.1038/s41416-018-0338-9 (PMC6265288; doi:10.1038/s41416-018-0338-9)
Supplement: Supplementary file 8 — Supplementary Table 4 [file 41416_2018_338_MOESM8_ESM.docx]

**Supplementary Table 5.** SOX9 and CK19 expression and the clinical outcome of iCCA patients that received chemotherapy

| **SOX9 IHC intensity** | **CK19 IHC intensity** | **Death or survival**  **(1=survival, 0=death)** | **Survival time**  **(months)** | **Survival time after CTx**  **(months)** | **Treatment** |
| --- | --- | --- | --- | --- | --- |
| low | low | 0 | 23 | 19 | Gemcitabine and XELOX |
| low | strong | 0 | 62 | 62* | Gemcitabine plus Cisplatin and XELOX |
| low | strong | 1 | 33 | 29 | Gemcitabine |
| low | low | 1 | 36 | 34 | Palliative chemotherapy |
| low | strong | 1 | 57 | 16 | Cisplatin and gemcitabine |
| low | low | 1 | 51 | 20 | Palliative chemotherapy |
| strong | strong | 0 | 14 | 13 | Erlotinib |
| strong | strong | 0 | 22 | 16 | Gemcitabine and cisplatin |
| strong | strong | 0 | 41 | 14 | Palliative chemotherapy |

CTx: Chemotherapy; XELOC: Capecitabine plus Oxaliplatin.

* The patient received chemotherapy following surgery.
