# Supplementary material for: SOX9 expression decreases survival of patients with intrahepatic cholangiocarcinoma by conferring chemoresistance
Source: Br J Cancer. 2018 Nov 13;119(11):1358–66. doi: 10.1038/s41416-018-0338-9 (PMC6265288; doi:10.1038/s41416-018-0338-9)
Supplement: Supplementary file 9 — Supplementary Table 5 [file 41416_2018_338_MOESM9_ESM.docx]

**Supplementary material**

**Supplementary Fig 1.** Representative IHC staining showing how to identify SOX9 intensity score.

**Supplementary Fig 2.** Knockdown of SOX9 alter gene and protein expression-associated with adenosine triphosphate–binding cassette transporters, drug metabolism enzymes and with p53 signalling in CC-SW-1 cells. (**A**) Microarray analysis (see Materials and Methods); (**B**) Western blot for MRP4 expression; (**C**) qPCR for ABCB1 gene expression.

**Supplementary Fig 3. Knockdown of SOX9 did not impact cisplatin-inhibited cell viability in CCA cells.** (**A-B**) MTT analyses showed cell viability in cisplatin-treated CC-SW1 and EGI-1 cells with or without SOX9 knockdown. (**C-D**) Phosphorylation of CHK1 and total CHK1 were measured in cisplatin-treated CC-SW1 and EGI-1 cells with or without SOX9 knockdown.

**Supplementary tables**

**Supplementary Table 1.** Clinicopathological features of patients with chronic liver disease

**Supplementary Table 2.** Clinicopathological features of validating set iCCA

**Supplementary Table 3**. Patient characteristics in iCCA patients with different levels of SOX9/CK19

**Supplementary Table 4.** Multivariate Analysis for Overall Survival of iCCA patients

**Supplementary Table 5.** SOX9 and CK19 expression and the clinical outcome of iCCA patients that received chemotherapy
